# Cholesterol neutralized vemurafenib treatment by promoting melanoma stem-like cells via its metabolite 27-hydroxycholesterol

**DOI:** 10.1007/s00018-024-05267-3

**Published:** 2024-05-22

**Authors:** Xiaohong Wang, Feiliang Zhong, Tingting Chen, Hongbo Wang, Weifang Wang, Hongkai Jin, Chouyang Li, Xuan Guo, Ying Liu, Yu Zhang, Bo Li

**Affiliations:** 1 Liaoning Technology and Engineering Center for Tumor Immunology and Molecular Theranotics, Collaborative Innovation Center for Age-Related Disease, Life Science Institute of Jinzhou Medical University, Jinzhou, 121001 Liaoning China; 2 College of Basic Medical Science, Jinzhou Medical University, Jinzhou, 121001 Liaoning China; 3 Key Laboratory of Industrial Fermentation Microbiology of the Ministry of Education, College of Biotechnology, Tianjin University of Science and Technology, Tianjin, 300457 People’s Republic of China; 4 School of Basic Medicine, Guangdong Medical University, Dongguan, 523808 Guangdong China

**Keywords:** DHCR24, Cholesterol metabolism, 27-Hydroxycholesterol, Melanoma

## Abstract

Vemurafenib has been used as first-line therapy for unresectable or metastatic melanoma with BRAFV600E mutation. However, overall survival is still limited due to treatment resistance after about one year. Therefore, identifying new therapeutic targets for melanoma is crucial for improving clinical outcomes. In the present study, we found that lowering intracellular cholesterol by knocking down DHCR24, the limiting synthetase, impaired tumor cell proliferation and migration and abrogated the ability to xenotransplant tumors. More importantly, administration of DHCR24 or cholesterol mediated resistance to vemurafenib and promoted the growth of melanoma spheroids. Mechanistically, we identified that 27-hydroxycholesterol (27HC), a primary metabolite of cholesterol synthesized by the enzyme cytochrome P450 27A1 (CYP27A1), reproduces the phenotypes induced by DHCR24 or cholesterol administration and activates Rap1-PI3K/AKT signaling. Accordingly, CYP27A1 is highly expressed in melanoma patients and upregulated by DHCR24 induction. Dafadine-A, a CYP27A1 inhibitor, attenuates cholesterol-induced growth of melanoma spheroids and abrogates the resistance property of vemurafenib-resistant melanoma cells. Finally, we confirmed that the effects of cholesterol on melanoma resistance require its metabolite 27HC through CYP27A1 catalysis, and that 27HC further upregulates Rap1A/Rap1B expression and increases AKT phosphorylation. Thus, our results suggest that targeting 27HC may be a useful strategy to overcome treatment resistance in metastatic melanoma.

**Supplementary Information:**

The online version contains supplementary material available at 10.1007/s00018-024-05267-3.

## Introduction

Malignant melanoma, which develops from the pigment cells of the skin mucosa, is the most serious skin cancer with high mortality. The V600E mutation of the B-Raf proto-oncogene, a serine/threonine kinase (BRAF), is the most common driver mutation in melanoma patients. It leads to constitutive activation of mitogen-activated protein kinase (MAPK), which promotes cell proliferation [1]. Although a number of important scientific milestones have been achieved in the fight against melanoma, the FDA approval of BRAF V600E and MEK inhibitors for example [2–5], the 5-year survival rate of metastatic individuals remains poor [6]. The surviving cancer stem cells (CSCs) contributed drug resistance is known to be the main issue for patients’ relapse [7, 8]. There is increasing evidence that cancer cells alter their cholesterol metabolism during tumorigenesis and treatment and that cholesterol and its metabolites contribute to CSC population by providing energy and proliferation signals [9–11]. Therefore, the identification of key mediators involved in metabolic dysregulation in specific contexts may provide novel therapeutic targets and highlight the importance of metabolic reprogramming in tumor biology.

In addition to chronic exposure to ultraviolet (UV) radiation from sunlight, obesity and type 2 diabetes have been shown to increase the risk of melanoma [12]. Gross and colleagues recently highlighted the link between UV exposure and metabolic disorders in melanoma biology by reporting a novel pathway in which UV radiation acts on cholesterol biosynthesis to control Ca2 + influx through Orai1, resulting in protein O-GlcNAcylation that promotes conversion to invasive melanoma [13]. DHCR24, 3β-Hydroxysterol Δ24-reductase, catalyzes the final step of cholesterol biosynthesis. Dysregulation of DHCR24 is associated with numerous diseases. Dhcr24 knockout mice died within hours of birth from a series of skin developmental defects related to desmosterol accumulation in the epidermis [14], and mutations in the DHCR24 gene lead to desmosterolosis, an autosomal recessive disease characterized by developmental and growth retardation [15]. As a key regulator of cellular cholesterol synthesis, DHCR24 was found to be downregulated in Alzheimer’s disease (AD) vulnerable regions and involved in AD-related pathological activities [16], and DHCR24 reverses AD-related pathology and cognitive impairment by increasing cholesterol levels in the hippocampus of 5xFAD mice [17]. In contrast, reduced expression of DHCR24 is associated with increased apoptosis in adrenocortical cells due to inhibition of caspase-3 [18]. More importantly, DHCR24 promotes the growth of CSC-like populations by activating the Hedgehog signaling pathway in breast cancer and protects melanoma cells from apoptosis [19–21], suggesting a possible role of DHCR24 or cholesterol in cancer metastasis and recurrence. However, DHCR24 is lower expressed in advanced tumors than in early tumors and normal tissues, and force expressed DHCR24 inhibits cell growth in prostate cancer [22, 23], suggesting that that cholesterol or its metabolites play a promoting or inhibitory role in tumorigenesis, depending on the type and stage of cancer [24, 25].

There are epidemiologic links between 27-hydroxycholesterol (27HC) and the progression of melanoma in obese or hypercholesterolemic individuals [26, 27]. 27HC is a primary metabolite of cholesterol synthesized by the enzyme cytochrome P450 27A1 (CYP27A1), and its role in breast and prostate cancer (PCa) has been extensively characterized [28, 29]. Nelson et al. found that the cholesterol metabolite 27HC promotes tumor growth and metastasis in mouse models of breast cancer by serving as a partial agonist for the estrogen receptor and liver X receptor [27]. In its role as a selective estrogen receptor modulator (SERM) [30, 31], 27HC has been shown to increase cell proliferation and modulate resistance to docetaxel-induced cytotoxicity in non-tumorigenic RWPE-1 prostate epithelial cells [32] and in LNCaP and PC3 cancer cells, 27HC increases cell proliferation by upregulating ERβ expression [32]. Although increasing CYP27A1, the rate-limiting enzyme in the formation of 27HC, led to a corresponding increase in the 27HC content of LNCaP and 22RV1 PCa cells and conditioned medium, the increase in 27HC content directly attenuated the proliferation of LNCaP and 22RV1-derived xenografts, by decreasing intracellular cholesterol as a consequence of attenuating SREBP2 activity [33], while the expression of CYP7B1, the rate-limiting enzyme in the degradation of 27HC, increases with the development and progression of PCa [34]. The differential effects of ERβ isoforms regulated by 27HC on EMT and metastasis in PCa may explain the equivocal role of 27HC. Similarly, it has been reported that hepatocyte-derived 27HC could stimulate ER in melanoma cells and control proliferation and differentiation via AKT and mitogen-activated protein kinase (MAPK) signaling pathways [21]. Indeed, there is an autoregulatory feedback loop to tightly regulate cholesterol homeostats. Physiologically, 27HC binds to insulin-induced proteins (INSIGs) to inhibit de novo cholesterol synthesis via SREBPs, and LXRs promote cholesterol efflux via ABCA1 and ABCG1, while decreasing uptake by downregulating LDL in the presence of excess cholesterol [35]. However, whether the feedback loop works in cancers that are drug resistant is not known.

In the present study, based on IHC results from clinical tissue microarray, DHCR24 was found to be highly expressed in melanoma patients, and knocking down DHCR24 blocked the cells in S phase and inhibited the proliferation and migration of melanoma cells. Next, we found that DHCR24 induced vemurafenib resistance in BRAF-mutated cells by promoting melanoma spheroid propagation. Targeted metabolomics analyses on DHCR24-inducing spheroids identified that 27HC was significantly upregulated upon DHCR24 expression, consistent with the fact that only CYP27A1 was highly expressed among cholesterol-degrading enzymes in the TCGA SKCM cohort. More importantly, in a model of drug resistance, we demonstrated that withdrawal of cholesterol or inhibition of CYP27A1 by dafadine-A abrogated the property of vemurafenib resistance (VR) cells and decreased Rap1A/Rap1B expression and AKT thr308/309 phosphorylation. Therefore, we concluded that our results have revealed a novel mechanism underlying cholesterol-mediated vemurafenib resistance in melanoma and proved that the cholesterol-27HC-Rap1 axis is a potential target for the treatment of vemurafenib-resistant melanoma.

## Materials and methods

### Cell lines and cell culture

A375 and 293T cells were obtained from Cell Resource Center of Peking Union Medical College (IBMS, CAMS/PUMC) and C1861 and VMM39 cells were purchased from Shanghai Fuyu Biotechnology. The A2058 melanoma cell was a kindly gift of Dr. Fang from the Beijing Institute of Genomics. Cells were grown in high-glucose DMEM medium (Gibco, 11995500BT) supplemented by 100 U/ml penicillin and 100 μg/ml streptomycin (TransGen Biotech), and 10% fetal bovine serum (FBS, Gibco, 10091-148) at 37 °C with 5% CO_2_.

### Immunohistochemistry on tissue microarrays (TMAs)

Skin cancer tissue Microarray (K063Me01) was purchased from Xi’an bioaitech Co., Ltd. (Xi’an, China). Protein expression was detected using IHC and analyzed according to standard method and microarray instruction. The TMAs were examined and scored independently by two pathologists. Tumor stages of the specimens on the tissue microarray were categorized according to the tumor-node-metastasis (TNM) system by American Joint Committee on cancer (AJCC) [36].

### Proliferation assay

Cell proliferation was quantified by Cell-counting kit-8 (CCK-8; TransGen, Beijing, China) following the manufacturer’s instructions. 6 μl CCK-8 kit solution was added to the medium after a total of 3 × 10^3^ cells seeded in each well of 96-well plates. The absorbance value (OD) was measured at 450 nm using a microplate reader (Thermo Fisher Scientific, Inc.). Each individual measurement was repeated three times.

### Cell cycle analysis

Flow cytometric analysis was performed to determine the effect of DHCR24 on cell cycle distribution. Briefly, cells grown in 6-well plates were treated with shRNA for 48 h. Then, cells were harvested and fixed in 75% ethanol solution. After centrifugation, cells were washed twice with cold PBS, then incubated with RNase A and stained with propidium iodide for 30 min in the dark. Cell cycle distribution was analyzed by flow cytometry (NovoCyte 2040R; ACEA Bioscience, Inc.; Agilent Technologies).

### Lentivirus packaging and infection

The lentivirus was packaged with the helper plasmids (PSPAX2, PMD2.G) by co-transfection into 293 T cells. The supernatant of the 293 T cells that packages lentivirus was harvested. The A375 and A2058 cells were infected with the lentivirus in the presence of 8 μg/ml polybrene. The cells were incubated at 37 °C in a CO_2_ incubator for 48 h, then selected with 1 μg/ml puromycin for 2 weeks.

### Construction of DHCR24 overexpression and shRNA constructs

The overexpression plasmid was constructed as following, gene sequences was amplified by reverse transcription-PCR using mRNA from human A2058 melanoma cells, the PCR product and pCDH (digested with EcoR1 and BamH1) fragments were then joined through Gibson assembly using NEBuilder HiFi DNA Assembly Master Mix (NEB). The DHCR24-specific shRNA sequences were chosen by BLOCK-iTTM RNAi Designer (http://rnaidesigner.thermofisher.com/rnaiexpress/).DHCR24 shRNA sequences are shRNA-6 (5′-CCGCGTGTGAAACACTTTGA-3′), shRNA-7 (5′-GCTCTCGCTTATCTTCGATA-3′), and control shRNA (shNC) (5′-GGTACGGTCAGGCAGCTTCT-3′). Short-hairpin sequences were synthesized as oligonucleotides and annealed according to standard protocol. Annealed shRNAs were then subcloned into pLL3.7 shRNA vectors (Addgene, #11795).

### Western blot analysis

Protein lysates from cells were extracted in RIPA buffer, with 1 × protease inhibitors cocktail. Equal number of proteins were resolved bySurePAGE™ Plus, Bis–Tris (GenScript, M00725) and transferred onto PVDF membranes. Blots were blocked and incubated with primary antibodies overnight at 4 °C. Then membranes were incubated with the secondary antibody at 37 °C for 1 h. The blots were probed with anti-DHCR24 (Santa Cruz Biotechnology, sc-398938), anti-Rap1A/Rap1B (Cell Signaling Technology, 2399), anti-AKT1 (Santa Cruz Biotechnology, sc-5298), anti-pAKT1-Thr308/309 (Signalway Antibody, 13311), anti-FLAG (ABCAM, ab205606), anti-CYP27A1 (ABCAM, ab126785), GAPDH (TransGen Biotech, HC301-01) were used as the internal control. Densitometry analysis was performed using ImageJ software.

### Transwell assay

The matrigel (ABW® Matrigengel, 082704) was diluted with medium (1:20), and spread evenly on the chamber for 1 h at 37 ℃. Then the cells suspended in serum-free medium were added to the upper chamber, and medium containing 10% FBS was filled in the lower chamber. After incubation for 24 h, the upper chamber was fixed in 70% methanol for 30 min. The images of chamber were captured and quantified after staining with 2.5% crystal violet. After removal of the cells on the upper membrane, the cells remained on the bottom membrane were identified as invasive cells.

### Subcutaneous tumor formation

Six-week-old NOD-SCID female mice were purchased from Beijing Vital River Laboratory Animal Technology Co., Ltd. (Beijing, China) and fed under SPF conditions in Institute of Radiation Medicine, Chinese Academy of Medical Science & Peking Union Medical College (Tianjin, China). NOD SCID female mice were injected subcutaneously with 1 × 10^7^ cells (0.1 ml PBS), then mice weighed twice a week. The volume of tumor was measured after three weeks, and tumor tissues were fixed in 4% paraformaldehyde for hematoxylin–eosin (HE) staining and Immunohistochemistry (IHC). Tumor volume size was calculated by the equation (length × width2/2).

### Real-time quantitative PCR analyses

Total RNA was prepared from cells using EasyPure® RNA Kit (TransGen Biotech) and reversely transcribed using TransScript® All-in-One First-Strand cDNA Synthesis SuperMix for qPCR (TransGen Biotech) according to the manufacturer’s protocol. Real-Time quantitative PCR was performed using SYBR SuperMix (TransGen Biotech) and specific primers are shown in Table 1.All the samples were normalized to the housekeeping gene, GAPDH. Data were calculated using the 2 − ΔΔCt method.

**Table 1:** Clinical-pathological information and TNM staging of human melanoma specimens (n = 63) used in this study

Subject	Gender	Age	Location	Pathology diagnosis	Grade	Stage	Type
A1	Female	45	Skin	Malignant melanoma of left chest wall	T3bN0M0	IIB	Malignant
A2	Female	62	Skin	Malignant melanoma of right foot	T4aN0M0	IIB	Malignant
A3	Male	45	Skin	Perianal malignant melanoma	T3bN0M0	IIB	Malignant
A4	Male	80	Skin	Malignant melanoma of right plantar	T4aN0M0	IIB	Malignant
A5	Female	46	Skin	Malignant melanoma of thigh	T4aN0M0	IIC	Malignant
A6	Male	42	Skin	Malignant melanoma of left heel	T3aN0M0	IIA	Malignant
A7	Female	74	Skin	Malignant melanoma of the back	T4aN0M0	IIB	Malignant
A8	Male	51	Skin	Malignant melanoma of the back	T4aN0M0	IIB	Malignant
B1	Male	37	Skin	Malignant melanoma of right upper arm	T4aN0M0	IIB	Malignant
B2	Male	61	Skin	Malignant melanoma of right groin	T4aN3M1a	IV	Malignant
B3	Female	47	Skin	Malignant melanoma of right calf sulcus	T4aN0M0	IIB	Malignant
B4	Female	66	Skin	Malignant melanoma of abdominal sulcus	T4bN0M0	IIC	Malignant
B5	Female	45	Skin	Malignant melanoma of left plantar	T4aN0M0	IIB	Malignant
B6	Female	35	Skin	Malignant melanoma of lumbar sulcus	T4aN3M1a	IV	Malignant
B7	Female	47	Skin	Malignant melanoma of right dorsum of foot	T4aN0M0	IIB	Malignant
B8	Male	76	Skin	Malignant melanoma of right anterior tibia	T4aN0M0	IIB	Malignant
C1	Female	72	Skin	Malignant melanoma of the left thigh root	T4aN0M0	IIB	Malignant
C2	Female	74	Skin	Malignant melanoma of left lower extremity	T4aN3M1a	IV	Malignant
C3	Male	63	Skin	Malignant melanoma of left occipital region	T4aN0M0	IIB	Malignant
C4	Male	61	Skin	Malignant melanoma deep in right lateral malleolus	T4aN0M0	IIB	Malignant
C5	Female	71	Skin	Malignant melanoma of the left medial thigh	T4aN0M0	IIB	Malignant
C6	Female	62	Skin	Malignant melanoma of left plantar	T4aN0M0	IIB	Malignant
C7	Male	61	Skin	Malignant melanoma of left plantar	T4aN0M0	IIB	Malignant
C8	Female	83	Skin	Malignant melanoma of left thumb	T4aN0M0	IIB	Malignant
D1	Male	71	Skin	Malignant melanoma of right buttock	T4aN0M0	IIB	Malignant
D2	Female	38	Skin	Malignant melanoma of right shoulder	T4aN1M0	III	Malignant
D3	Male	49	Skin	Malignant melanoma of left thigh	T4aN0M0	IIB	Malignant
D4	Female	42	Skin	Malignant melanoma of right thigh	T4aN0M0	IIB	Malignant
D5	Male	7	Skin	Malignant melanoma of sacrococcygeal region	T4aN0M0	IIB	Malignant
D6	Female	70	Skin	Malignant melanoma of left parotid gland	–	–	Malignant
D7	Male	50	Esophagus	Malignant melanoma of esophagus	–	–	Malignant
D8	Female	62	Urethra	Malignant melanoma of urinary tract	–	–	Malignant
E1	Female	47	Cavidade nasal	Malignant melanoma of nasal cavity	–	–	Malignant
E2	Male	63	Cavidade nasal	Malignant melanoma of left maxillary sinus	–	–	Malignant
E3	Female	48	Mediastinum	Malignant melanoma of mediastinum	–	–	Malignant
E4	Male	46	Skin	Malignant melanoma of scrotum	T4aN0M0	IIB	Malignant
E5	Male	62	Skin	Malignant melanoma of scrotum	T4aN0M0	IIC	Malignant
E6	Male	37	Eye	Malignant melanoma of choroid	T3N1M0	IV	Malignant
E7	Female	46	Lymph node	Metastatic malignant melanoma of groin	–	–	Metastasis
E8	Male	45	Lymph node	Right axillary metastatic malignant melanoma	–	–	Metastasis
F1	Female	40	Lymph node	Metastatic malignant melanoma of maxilla	–	–	Metastasis
F2	Female	61	Lymph node	Metastatic melanoma of right groin	–	–	Metastasis
F3	Male	65	Lymph node	Metastatic malignant melanoma of right groin	–	-	Metastasis
F4	Female	48	Lymph node	Metastatic malignant melanoma of mediastinum	–	–	Metastasis
F5	Male	73	Small intestine	Metastatic malignant melanoma of small intestine	–	–	Metastasis
F6	Male	70	Lymph node	Metastatic malignant melanoma of axilla	–	–	Metastasis
F7	Female	51	Liver	Metastatic malignant melanoma of right lobe of liver	–	–	Metastasis
F8	Male	49	Lymph node	Left axillary metastatic malignant melanoma	–	–	Metastasis
G1	Female	56	Skin	Skin	–	–	Control
G2	Male	35	Skin	Skin	–	–	Control
G3	Male	28	Skin	Skin tissue of scalp	–	–	Control
G4	Female	40	Skin	Skin tissue of scalp	–	–	Control
G5	Female	42	Skin	Skin tissue of scalp	–	–	Control
G6	Male	30	Skin	Skin tissue of scalp	-	-	Control
G7	Male	40	Skin	Abdominal skin tissue	–	–	Control
G8	Male	36	Oral cavity	Pharyngeal mucosa and skin tissue	–	–	Control
H1	Male	48	Oral cavity	Pharyngeal mucosa and skin tissue	–	–	Control
H2	Male	28	Esophagus	Esophageal mucosa	–	–	Control
H3	Female	40	Esophagus	Esophageal mucosa	–	–	Control
H4	Male	45	Small intestine	Small intestinal mucosa	–	–	Control
H5	Male	40	Small intestine	Small intestinal mucosa	–	–	Control
H6	Male	30	Lymph node	Axillary lymph node tissue	–	–	Control
H7	Female	33	Lymph node	Cervical lymph node tissue	–	–	Control

### Rhodamine transport assay

The cell lines were incubated with 15 μM Rhodamine 123 (MedChemExpress, HY-D0816) at 37 °C for 5 min and washed three times with the culture medium. Afterwards, cells were incubated in the culture medium with varying lapatinib concentrations at 37 °C for 30 min. The cell lines were then analyzed by flow cytometry. (BD Biosciences, San Jose, CA, USA).

### Flow cytometry analysis

For cell cycle assay, the cells were fixed with 70% ethanol at 4 °C overnight. The cells were added RNase A (100 μg/ml) for 5 min at room temperature, then stained with PI (50 μg/ml, US EVERBRIGHT, Y6002) for 30 min at room temperature. All samples were detected using a flow cytometer (NovoCyte 2040R; Agilent Technologies) and analyzed using the NovoExpress 1.4.1 software (Agilent Technologies).

### 27HC and cholesterol determination

Human 27HC ELISA Kit was obtained from Wuhan Fine Biotech Co., Ltd (Wuhan, China). Cells (2 × 10^6^) were collected and detected for 27HC content by Human 27HC ELISA Kit following manufacturer’s protocol. The OD value was measured by a Microplate Reader (Thermo) at 450 nm. The concentration of cellular 27HC was obtained according to standards.

Cholesterol was measured with the Amplex Red cholesterol assay kit (Thermo Fisher Scientific, A12216) according to the manufacturer’s instruction. Measured the fluorescence in a fluorescence microplate reader using excitation at 530 nm and emission detection at 590 nm. The concentration of cellular cholesterol was obtained according to standards.

### Membrane fluidity assay

The membrane fluidity of cells was measured using the lipophilic pyrene probe, a lipid analogue probe that underwent excimer formation upon spatial interaction with the cellular membrane (Membrane Fluidity Kit, Abcam, #ab189819). Cells were treated for 48 h, followed by 1 h incubation with 2 µM PDA and 0.08% pluronic F-127. PDA fluorescence was monitored by exciting at 350 nM and taking emission values at 400 nM (monomer) and 470 nM (excimer). Relative membrane fluidity is a ratio of excimer to monomer fluorescence (Ie/Im).

### Determination of cholesterol metabolites

1 × 10^7^ cells were collected and added 400 μl methanol: water: chloroform (5:2:2, v/v/v) mixed solution, then pre-cool at −20 °C for 20 min. The samples were homogenized in a micro-homogenizer for 3 min, and extracted ultrasonically for 2 times (10 min each time) in an ice-water bath. The supernatant was collected at 13,000 rpm (4 ℃) for 10 min and concentrated with a centrifugal concentration dryer. Dried lipids were dissolved with 200 μl methanol: water (4:1, v/v) mixture solution and vortex for 30 s or ultrasonicate in ice water bath until mixture was homogenous. Filtering with 0.22 μm organic phase pinhole filter for LC–MS analysis.

Liquid chromatography was performed using an UPLC system (Nexera UHPLC LC-30A). The chromatographic column was the Waters UPLC HSS C18 Column (2.1 mm × 100 mm, 1.7 µm). The mobile phase A was water containing 0.1% formic acid, and the mobile phase B was acetonitrile. The temperature of column was 45 °C, and the flowing rate of mobile phase was 0.4 ml/min. Gradient conditions were as follows: 0–0.5 min, 40 B; 0.5–1 min, 40%-55%B; 1–8 min, 50%-90% B; 8–9.2 min, 90%–40% B; 9.2–10 min, 40% B. Mass spectrometry analyzed using an AB Sciex Qtrap 5500 system containing an electrospray ionization (ESI) source. The capillary voltage was set to 5500 V for positive mode and 4500 V for negative mode. The mass spectra were acquired in a mass range of m/z 100–1000, and collision-activated dissociation (CAD) was medium. Other parameters were as follow: ion source gas 1, 60 psi; ion source gas 2, 50 psi; curtain gas (CUR), 35 psi; and turbo ion spray source temperature, 500 °C.

All standards were purchased from Sigma and prepared at the concentrations of 0.05 ng/ml, 0.13 ng/ml, 0.33 ng/ml, 0.82 ng/ml, 2.05 ng/ml, 5.12 ng/ml, 12.80 ng/ml, 32.00 ng/ml, 80.00 ng/ml, 200.00 ng/ml, 500.00 ng/ml and 1000.00 ng/ml. The content of cholesterol and its metabolites in different cells were obtained according to standards.

### Cell morphological analysis

Cell morphological was on showed with Wright-Giemsa Stain Kit (Solarbio, G1021) according to the manufacturer’s instruction. Observation was made under inverted fluorescence microscope.

### Cancer spheroid assay

For spheroid assay, single cell suspensions harvested and seeded in 6-well ultralow adherent cell culture plates, at a density of 1000 cells/ml in serum-free DMEM/F12 medium supplemented with 1% L-glutamine, 1% penicillin/streptomycin, 2% B27 (Invitrogen, Carlsbad, CA), 20 ng/ml epidermal growth factor (EGF, Sigma, St. Louis, MO) and 20 ng/ml basic fibroblast growth factor (bFGF, Invitrogen, Carlsbad, CA). 7 days after seeding, spheroids with diameter > 30 μm were counted using an inverted microscope (× 100) and the number of colony formation was calculated. Each experiment was repeated twice with consistency.

### Histology and morphometric analysis

Tumors were collected and fixed in 10% neutral-buffered formalin. Tissues were sectioned and stained with hemoxylin and eosin (HE). Pictures were observed under an optical microscope (BX51, Olympus, Tokyo, Japan) to evaluate pathological morphology.

### Whole-transcriptome sequencing

Total RNA was extracted and lysed in 500 μl TRIzol reagent (MRC, Carrollton, OH) and sent to China’s Shenzhen based BGI (Shenzhen, China) for further analysis. The RNA-seq library was created using the Illumina TruSeq RNA Sample preparation Kit v2 with standard protocol. Genes with a *P* adjusted value (false-discover rate) < 0.05 were selected for the Gene Ontology (GO) analysis and for the heatmap. Pathway analysis was performed using the Kyoto Encyclopedia of Genes and Genomes (KEGG) database.

### Statistics

Statistical analyses were performed using Prism 8 software (GraphPad, La Jolla, CA, USA). For comparison between two groups, a two-tailed Student t-test was used. Kaplan–Meier survival analysis was performed for the comparison of survival curves. The statistical significance of protein associations in the TMA data set was evaluated using the Pearson chi-square test. Statistically significant levels were defined as ns (not significant, *P* > 0.05), * *P* < 0.05, ** P < 0.01, *** P < 0.001. All data were presented as mean ± SD.

## Results

### DHCR24 is upregulated in melanoma and is essential for cancer cell survival

**Fig.1:**
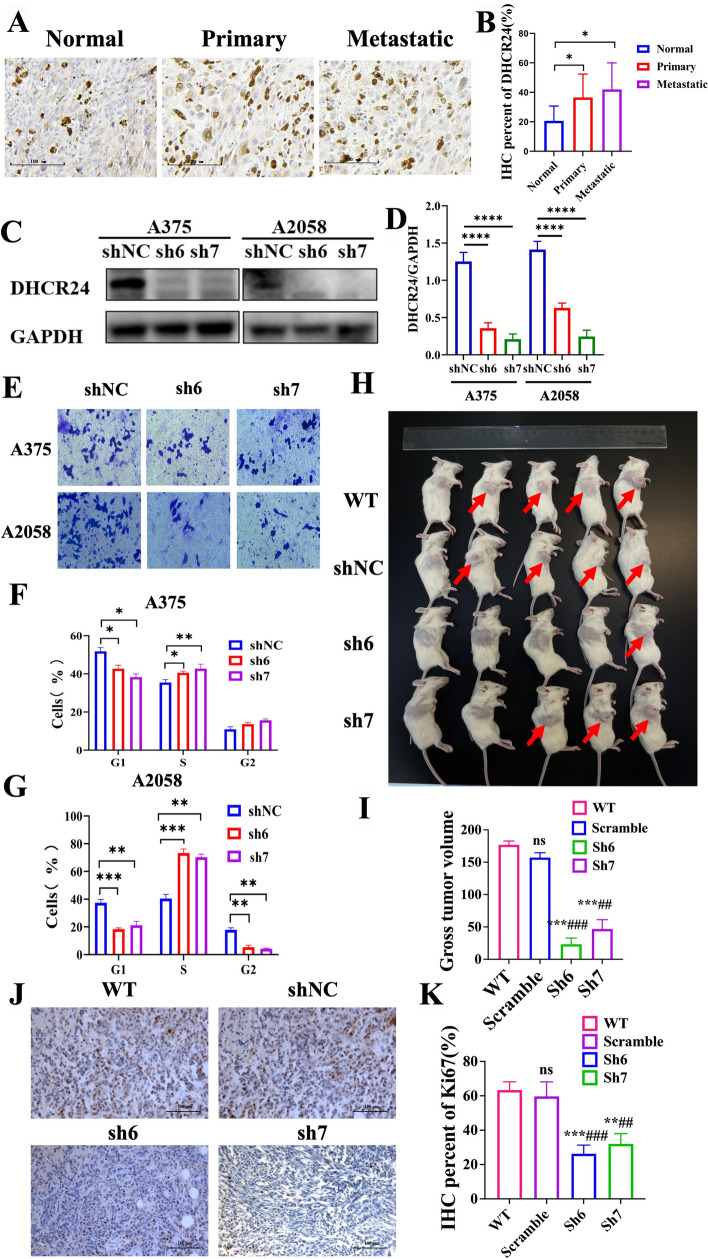
DHCR24 is highly expressed in melanoma and essential for tumor growth. **A**, **B** Representative images and quantitative bar chart of immunohistochemistry staining against DHCR24 in normal skin tissue, primary and metastatic malignant melanoma samples. **C**, **D** Western blot and quantitative bar chart of DHCR24 knock down efficiency in melanoma cell lines using lentivirus harboring scramble shRNA (shNC) and DHCR24 shRNAs (sh6 and sh7). **E** Transwell assay of A375 and A2058 melanoma cells harboring scramble shRNA (shNC) and DHCR24 shRNAs (sh6 and sh7). **F**, **G** Cell cycle assay determined by flow cytometry of A375 and A2058 melanoma cells harboring scramble shRNA (shNC) and DHCR24 shRNAs (sh6 and sh7). **H** NOD-SCID mice were subcutaneously injected with 1 × 10^7^ sh6 and sh7 A375 cells (n = 5) compared with the wild type and shNC groups (n = 5 each). **I** The tumor volume among four groups. **J**–**K** Representative images and quantitative bar chart of immunohistochemistry staining against Ki67 in xenograft tumor tissues. Different colors represent different groups, *p < 0.05, **p < 0.01, ***p < 0.001, asterisks (*) stand for significance levels

To investigate the role of DHCR24 in melanoma tumorigenesis, immunohistochemical (IHC) staining was performed using tissue chip containing normal skin tissue (n = 15), primary melanoma (n = 38) and metastatic melanoma patient samples (n = 10) (see Supplementary Table 1 for details). The results showed that the expression of DHCR24 was significantly higher in malignant melanoma, especially in metastatic melanoma, compared with control (Fig. 1A, B). Subsequently, by using two different shRNA constructs targeting DHCR24 (Fig. 1C, D), we found that knockdown of DHCR24 significantly slowed cell proliferation at 48–96 h (Fig. S1A) and significantly decreased the invasion and migration ability of the knockdown group compared with the control group (Fig. 1E). Flow cytometry analysis showed that knockdown of DHCR24 decreased the number of cells in the G1 phase, while the number of cells in the S phase increased (Fig. 1F–G), indicating cell cycle arrest. To illustrate the effect of DHCR24 on tumorigenic capacity in vivo, a xenograft assay was performed in mice with DHCR24 knockdown and overexpression of melanoma cells. In the knockdown group, tumor volume decreased significantly (Fig. 1H–I), which was consistent with IHC staining Ki67 (Fig. 1J–K) after 21 days of injection. Conversely, overexpression of DHCR24 resulted in a dramatic increase in tumor volume but no significant change in body weight (Fig. S1C, D). These data indicate that DHCR24 plays an oncogenic role in melanoma cell survival and migration.

### DHCR24 is associated with vemurafenib resistance and promotes melanoma spheroid propagation

**Fig. 2:**
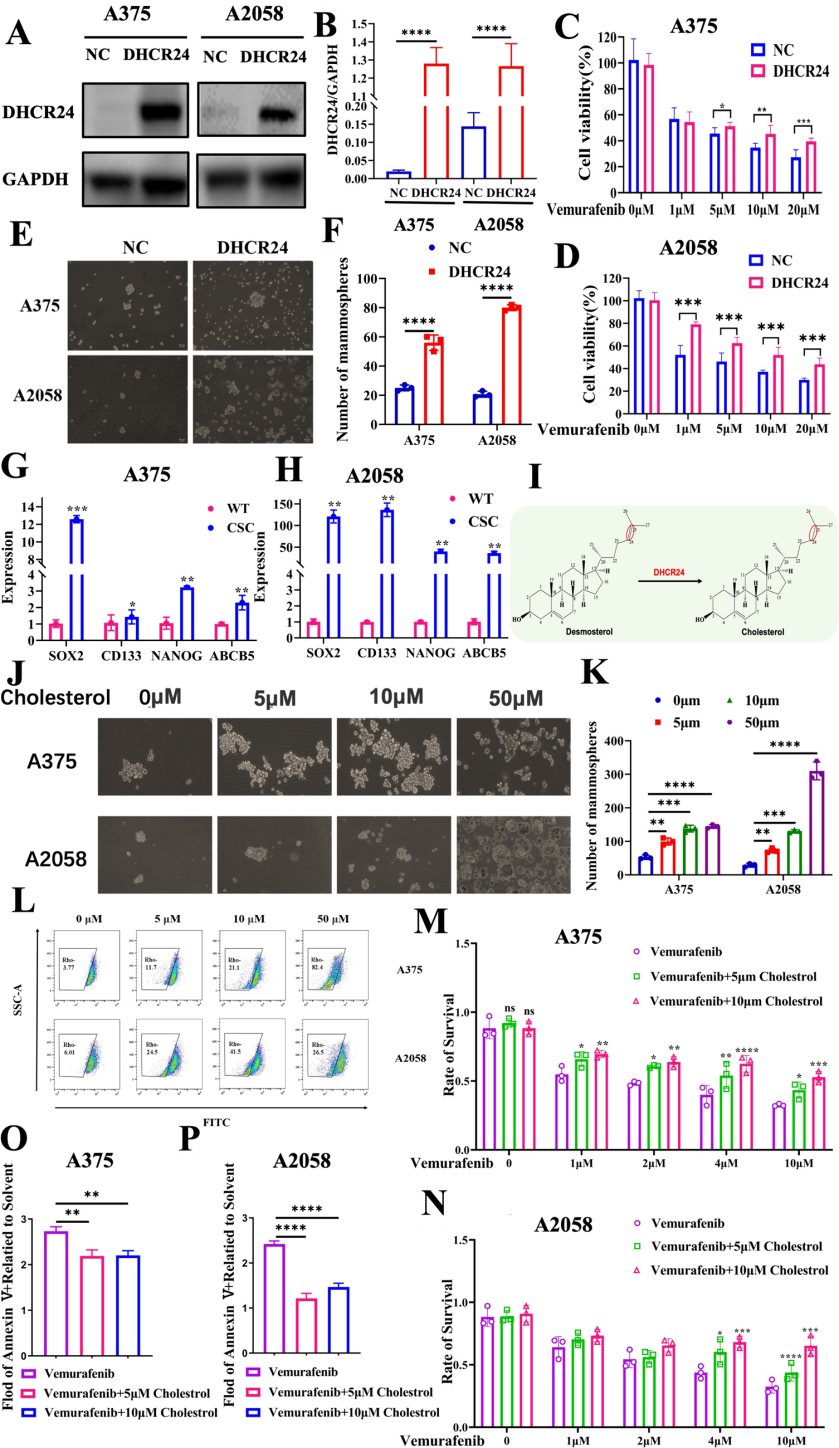
DHCR24 is associated with vemurafenib resistance and promotes melanoma spheroid propagation. **A**, **B** Western blot and quantitative bar chart of DHCR24 overexpression in melanoma cell lines. **C**, **D** Percentage of melanoma cell viability under the treatment of vemurafenib (1, 5, 10 and 20 μM) in presence of DHCR24 overexpression or not for 48 h determined by CKK-8. **E**, **F** Representative images and quantitative bar chart of spheroid propagation in melanoma cells with DHCR24 overexpression. **G**, **H** The stemness of spheroid is evaluated by stem markers (Sox2, CD133, Nanog and ABCB5) mRNA expression. **I** Chemical equation of cholesterol synthesis from desmosterol catalyzed by DHCR24, which reduces the double bond at C24–C25 position of desmosterol. **J**, **K** Representative images and quantitative bar chart of spheroid propagation in A375 and A2058 in presence of cholesterol with gradient concentration. **L** The stemness of spheroid is evaluated by Rhodamine 123. **M**, **N** Percentage of melanoma cell viability under vemurafenib treatment (1, 2, 4 and 10 μM) in presence of cholesterol (5 and 10 μM) or not for 48 h. **O**–**P** Apoptosis rate of melanoma cell viability under vemurafenib treatment (10 μM) in presence of cholesterol (5 and 10 μM) or not for 48 h determined by flow cytometry

To further investigate the function of DHCR24 in melanoma therapy, the proliferation assay was performed under vemurafenib treatment in DHCR24-expressing melanoma cells (Fig. 2A, B). The result showed that DHCR24 partially rescued the proliferation blockade caused by vemurafenib (Fig. 2C, D). Using an in vitro tumor sphere formation assay, we found that DHCR24 contributed to the proliferation of stem-like melanoma cell populations (Fig. 2E, F), and the stem cell property of the spheroids was confirmed by the expression of Sox2, CD133, Nanog and ABCB5 (Fig. 2G–H). Considering that DHCR24 is the final enzyme of the cholesterol biosynthetic pathway that converts desmosterol to cholesterol by catalyzing the reduction of C24 = C25 unsaturation in the side chain (Fig. 2I). Therefore, we next treated the A375 and A2058 melanoma cells with a gradient of concentrated cholesterol (0, 5, 10, 50 μM) on the poly-HEMA-coated 12-well plates under tumor sphere culture conditions for 7–10 days. As shown in Fig. 2F, the number and size of spheroids in melanoma cells increased significantly after cholesterol administration compared to the solvent control. In addition, rhodamine123 staining showed that more “negative” cell populations appeared in two BRAF-V600E-mutated melanoma cells with increasing cholesterol levels (Fig. 2J–L). Next, we validated that cholesterol rescued vemurafenib-induced proliferation blockade and apoptosis (Fig. 2M–P) as DHCR24 forced expression, highlighting the importance of cholesterol in melanoma therapy.

### DHCR24 induces Rap1-AKT activation and cellular 27HC accumulation

**Fig.3:**
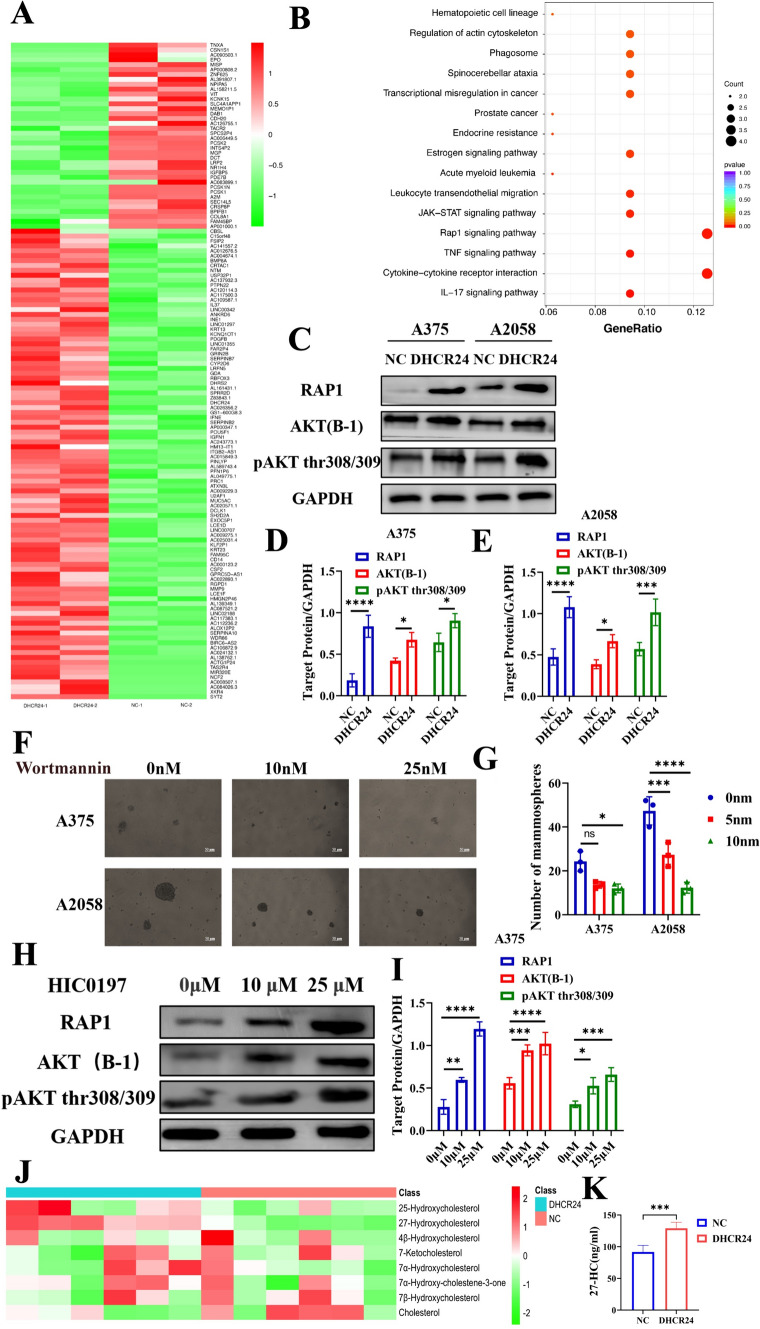
DHCR24 activates Rap1-AKT signaling induces cellular 27HC accumulation. **A** Heatmap of spheroids induced by DHCR24 overexpressed A375 cell (134 genes, FDR-corrected P value < 0.05 and ≥ twofold change cut-off), totally 38 downregulated genes and 96 upregulated genes. **B** KEGG enrichment analysis (representative pathways) of 96 upregulated genes in A375 spheroids with DHCR24 overexpression. **C**–**E** Western blots and quantitative bar chart of Rap1A/Rap1B, AKT and pAKT in spheroids induced by DHCR24 overexpressed A375 and A2058 cells. **F**–**G** Representative images and quantitative bar chart of spheroid propagation of A375 and A2058 melanoma cells with wortmannin (PI3K inhibitor) treatment. **H**, **I** Western blots and quantitative bar chart of Rap1A/Rap1B, AKT and pAKT in A375 melanoma cell treated by Rap1 agonist HJC0197. **J** Heatmap of 9 paneled cholesterol metabolites quantitation of melanoma cells with DHCR24 overexpression. **K** Cellular 27HC quantitation in melanoma cells with DHCR24 overexpression determined by ELISA. ***p < 0.001, asterisks (*) stand for significance levels

To decipher the molecular mechanism underlying DHCR24, spheroids induced by DHCR24 and a control group were subjected to whole transcriptome sequencing. The heatmap showed that 134 genes were differentially expressed, including 38 down-regulated genes and 96 up-regulated genes (fold change ≥ 2 and a P value (t-test) < 0.05) (Fig. 3A). KEGG enrichment analysis of 96 upregulated genes demonstrated that the Rap1 signaling pathway, which is thought to be upstream of the PI3K/AKT signaling pathway [37], was significantly enriched in DHCR24-induced melanoma spheroids (Fig. 3B). Western blot results showed that Rap1A/ Rap1B expression and AKT phosphorylation (thr 308/309) were significantly increased (Fig. 3C–E), and the PI3K inhibitor wortmannin markedly attenuated spheroids under tumor sphere culture conditions (Fig. 3F–G), confirming that PI3K/AKT activation contributes to melanoma stem cell formation. To investigate the relationship between Rap1 and AKT signaling, HIC0197 was used to activate Rap1 signaling and then AKT phosphorylation was determined by Western blot. The results demonstrated that both AKT and AKT phosphorylation were upregulated with the increase in Rap1A/ Rap1B expression (Fig. 3H, I). Therefore, we hypothesized that Rap1-AKT signaling is downstream of DHCR24 regulation and positively related to cancer stem cells. Although DHCR24 is the final limiting enzyme of cholesterol synthesis, the catabolic fate of cholesterol in melanoma cells was not yet understood. Therefore, the profile of 17 metabolites downstream of cholesterol was characterized by LC–MS after forced DHCR24 expression. In melanoma cell A2058, 8 of 17 metabolites, including cholesterol, were detected and quantified. Among these metabolites, 27-hydroxycholesterol (27HC) was increased by 41.39%, which was statistically significant (n = 6, p = 0.0000751, see Table 2 and Fig. 3J). The increase in 27HC was further verified using a human 27HC ELISA kit (Fig. 3K). These results indicated that 27HC might be final effector of DHCR24 induced vemurafenib resistance and tumor spheroid propagation.

**Table 2:** Qualitative and quantitative detection of cholesterol metabolites by LC–MS in the melanoma cell with overexpressed DHCR24

Compound	Average (NC) (ng/ml)	Average (DHCR24) (ng/ml)	FC (DHCR24/NC)	*p* Value
27-Hydroxycholesterol	89.784	129.240	1.439	0.0000751
25-Hydroxycholesterol	2.338	3.108	1.330	0.1311811
7α-Hydroxycholesterol	13.642	14.948	1.096	0.3981013
7a-Hydroxy-cholestene-3-one	2.486	2.700	1.086	0.4755491
7β-Hydroxycholesterol	17.241	16.509	0.958	0.6148834
7-Ketocholesterol	35.229	33.715	0.957	0.5765399
Cholesterol	6211.131	5676.205	0.914	0.0951267
4β-Hydroxycholesterol	5.561	4.991	0.897	0.7118280
Prostaglandin E2	0.141	0.116	0.827	0.2729710
22β-Hydroxycholesterol	0.000	0.000	0.000	0.0000000
24(S)-hydroxycholesterol	0.000	0.000	0.000	0.0000000
Aldosterone	0.000	0.000	0.000	0.0000000
Androsterone	0.000	0.000	0.000	0.0000000
Dehydroepiandrosterone sulfate	1.114	0.000	0.000	0.3632175
Deoxycorticosterone	0.000	0.000	0.000	0.0000000
Progesterone	0.000	0.000	0.000	0.0000000
Tetrahydrocorticosterone	0.000	0.000	0.000	0.0000000

### 27HC promotes spheroid propagation in melanoma and increases vemurafenib resistance

**Fig. 4.**
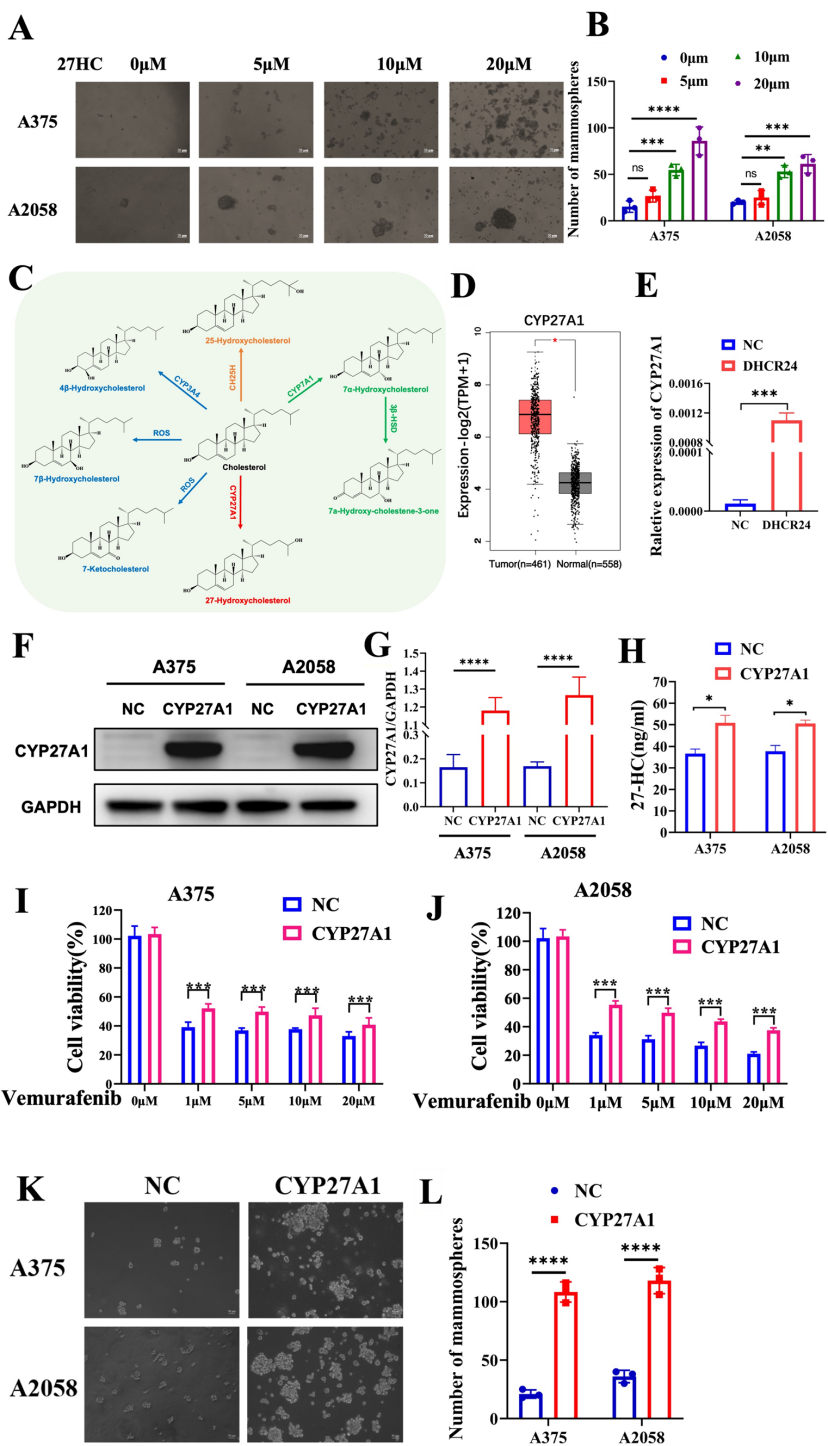
27HC promotes melanoma spheroids propagation and increases vemurafenib resistance. **A**, **B** Representative images and quantitative bar chart of spheroid propagation of A375 and A2058 in presence of 27HC with gradient concentration. **C** Diagram of cholesterol centered metabolic pathway. **D** Expression levels of CYP27A1 in Skin Cutaneous Melanoma (SKCM) samples compared with normal tissue, the RNA-Seq datasets is based on the UCSC Xena project (http://xena.ucsc.edu), and visualized by GEPIA (http://gepia.cancer-pku.cn). **E** DHCR24 induces CYP27A1 mRNA upregulation in A2058 melanoma cells. **F**–**G** Western blot and quantitative bar chart of CYP27A1 overexpression in A375 and A2058 cells. **H** Cellular 27HC quantitation in melanoma cells with CYP27A1 overexpression determined by ELISA. **I**, **J** Percentage of melanoma cell viability under the treated with vemurafenib (1, 5, 10 and 20 μM) in presence of CYP27A1 overexpression or not for 48 h determined by CKK-8. **K**–**J** Representative images and quantitative bar chart of spheroid propagation in melanoma cells with CYP27A1 overexpression. *p < 0.05, ***p < 0.001, asterisks (*) stand for significance levels

To validate the role of 27HC in melanoma, a tumor spheroid assay was also performed on wild-type melanoma cells treated with gradient concentrations (0, 5, 10 and 20 μM) of 27HC. As shown in Fig. 4A, B the number and size of tumor spheroids were significantly increased in A375 and A2058 melanoma cells after 7–10 days of treatment with 27HC compared to solvent (ethanol as control). Next, we examined the genes encoding enzymes involved in the cholesterol-centered metabolic pathway (Fig. 4C). Only CYP27A1, encoding 27-hydroxylase, which catalyzes the conversion of cholesterol to 27HC, was highly expressed in melanoma patients compared to normal patients and melanocytes according to the TCGA-SKCM cohorts (Fig. 4D). We also found that DHCR24 strongly upregulated the expression of CYP27A1 in melanoma cells (Fig. 4E), indicating a positive feedback loop leading to cellular accumulation of 27HC. Accordingly, forced expression of CYP27A1 only in melanoma cells (Fig. 4F–G) increased cellular 27HC content (Fig. 4H), caused vemurafenib resistance (Fig. 4I, J), and promoted melanoma spheroid propagation (Fig. 4K, L) in both A375 and A2058 melanoma cells, independent of DHCR24 induction. These data confirm that 27HC as the main downstream effector of DHCR24 promotes melanoma spheroid propagation and increases vemurafenib resistance.

### Depletion of 27HC reduces melanoma spheroids and improves treatment with vemurafenib

**Fig. 5.**
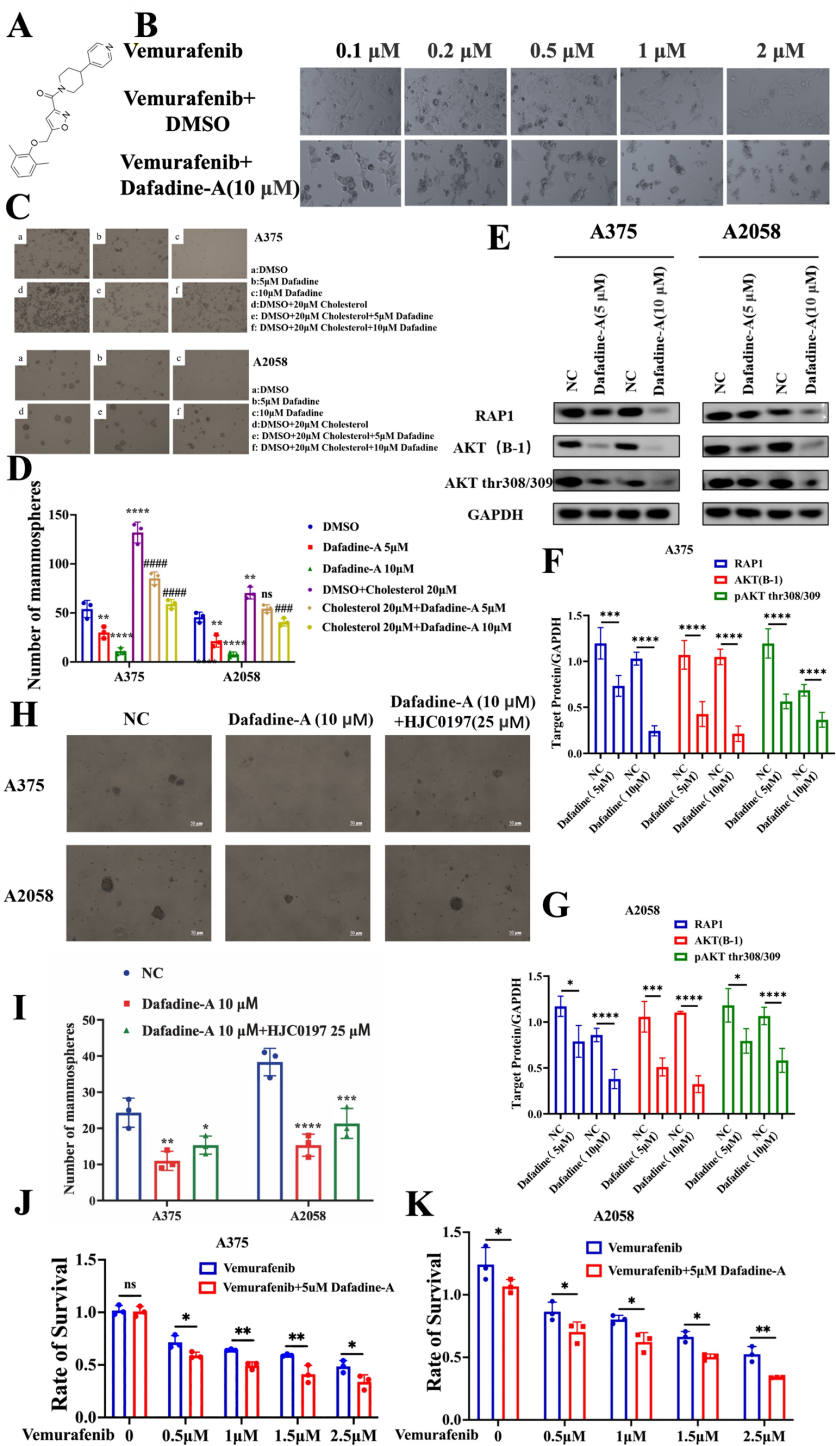
27HC depletion decreases melanoma spheroids and enhances vemurafenib treatment. **A** Chemical formula of dafadine-A (CYP27A1 inhibitor). **B** Morphological images of A375 melanoma cells were treated with vemurafenib alone or combinational use with dafadine-A for 48 h. **C**, **D** Dafadine-A alone or combinational use with cholesterol (20 μM) on A375 and A2058 melanoma cells for evaluating the contribution of dafadine-A on melanoma spheroid f propagation. **E**–**G** Western blot and quantitative bar chart of Rap1A/Rap1B, AKT and pAKT in A375 and A2058 melanoma cells with dafadine-A treatment. **I** Dafadine-A alone or combinational use with HJC0197 (25 μM) on A375 and A2058 melanoma cells for evaluating the contribution of Rap1 downstream 27HC on melanoma spheroid propagation. **J**, **K** Dafadine-A combinational use with vemurafenib on A375 and A2058 for 48 h determined by CKK-8

Next, we sought to determine the effect of dafadine-A, a novel inhibitor of DAF-9 cytochrome P450 in the nematode Caenorhabditis elegans and the mammalian ortholog of DAF-9 (CYP27A1), on melanoma cells (Fig. 5A). We found that dafadine-A did not affect the proliferation of adherent melanoma cells (Fig. 5B), but dramatically inhibited cholesterol-induced melanoma spheroid propagation (Fig. 5C, D), with downregulation of Rap1A/Rap1B expression and phosphorylated AKT1-thr308/309 (Fig. 5E–G). Accordingly, HIC0197 was able to abrogate the inhibition of melanoma spheroid propagation induced by dafadine-A (Fig. 5H, I), confirming that Rap1 is functional upstream of AKT signaling. When used in combination, we observed that dafadine-A significantly enhanced vemurafenib-induced apoptosis (Fig. 5J, K). These data emphasized the indispensable role of CYP27A1 in melanoma stemness and the potential targeting property.

### Cholesterol metabolism is a promising therapeutic target for vemurafenib-resistant melanoma

**Fig. 6.**
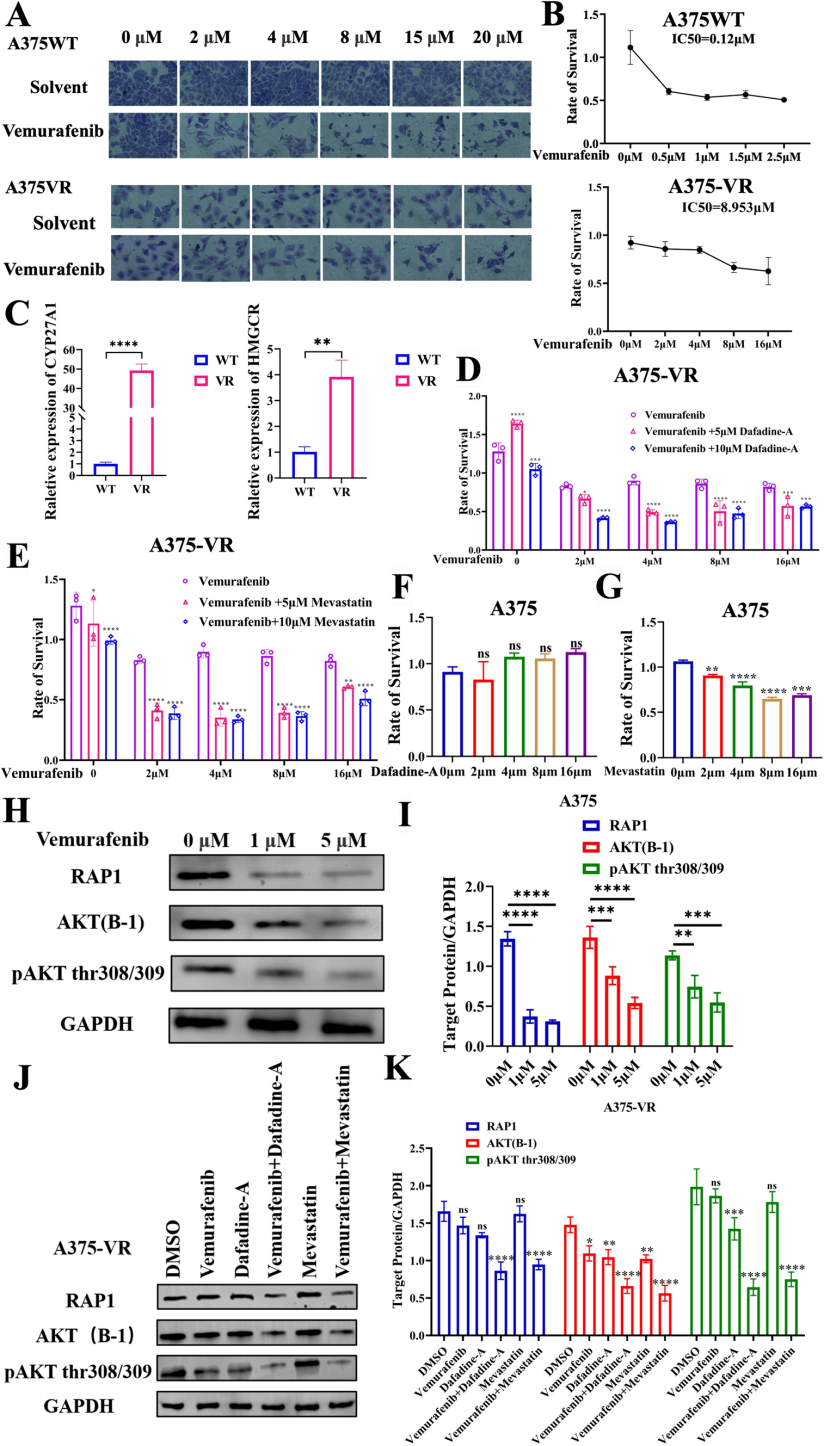
27HC is promising therapeutic target of vemurafenib resistant melanoma*.* **A** Morphological images of A375 and A375 vemurafenib resistant melanoma cell (A375VR) treated by gradient concentration of vemurafenib (2, 4, 8,15 and 20 μM). **B** IC50 determination of vemurafenib on A375 and A375VR cells. **C** The mRNA expression level of CYP27A1 and HMGCR in A375 and A375VR cells. Combinational use vemurafenib with **D** Dafadine-A or **E** Mevastatin on A375VR cells. **F**–**G** Percentage of A375 cell viability under the treatment of dafadine-A or mevastatin for 48 h determined by CKK-8. **H**, **I** Western blot and quantitative bar chart of Rap1A/Rap1B, AKT and pAKT in A375 cells under the treatment of vemurafenib. **J**, **K** Western blot and quantitative bar chart of Rap1A/Rap1B, AKT and pAKT in A375VR cells with combinational treatment vemurafenib and dafadine-A or mevastatin. *p < 0.05, **p < 0.01, ***p < 0.001, ****p < 0.0001, asterisks (*) stand for significance level

To further investigate the potential role of targeted therapy by inhibiting the cholesterol-27HC-Rap1 axis in vemurafenib-resistant melanoma, a vemurafenib-resistant A375 cell line (A375-VR) was established by continuous treatment with vemurafenib for weeks. The adaptive resistance of A375-VR was confirmed by Giemsa staining under serial vemurafenib treatment compared to the A375 wild-type counterpart (Fig. 6A), and the IC50 was calculated by CKK8 determinations (Fig. 6B). The value of A375-VR was almost 75-fold higher than that of parental A375 cells (8.593 μM vs. 0.12 μM), demonstrating the vemurafenib-resistant property of A375-VR cells. Interestingly, we found that A375-VR cells exhibited higher expression of CYP27A1 and the cholesterol synthesis-limiting enzyme HMGCR than the parental cells (Fig. 6C). Dafadine-A and mevastatin (HMGCR inhibitor) were therefore used in combination with vemurafenib. Compared to the moderate decrease induced by vemurafenib, dafadine-A and mevastatin dramatically abrogated the inhibitory effects mediated by vemurafenib (Fig. 6D, E), while A375 VR cells proliferation was unaffected (Fig. 6F, G). Mechanistically, after the addition of vemurafenib, the expression of Rap1A/Rap1B and phosphorylated AKT1-thr308/309 was decreased in wild-type cells (Fig. 6H, I), whereas the expression of Rap1A/Rap1B and phosphorylated AKT1-thr308/309 was not altered in A375-VR cells, but was decreased in cells receiving vemurafenib and dafadine-A or mevastatin in combination (Fig. 6J, K).

## Discussion

The persistent activation of oncogenic signals enables cancer cells to synthesize their own cholesterol to provide energy for rapid growth. Accordingly, numerous studies show that statins, inhibitors of HMG-CoA reductase (HMGCR), directly delay cancer development and progression in cell and animal models [38, 39]. In addition, survival analyzes using the Cancer Genome Atlas (TCGA) show that increased activity of the cholesterol synthesis pathway correlates with lower patient survival in sarcoma, acute myeloid leukemia and melanoma [40]. By directly regulating signaling molecules in the membrane [41, 42], or as signaling molecules via certain metabolites [43], cholesterol has a significant impact on the biological behavior of cancer and promotes or suppresses cancer development, depending on the cell context and the steps of metabolic perturbation [44, 45]. Therefore, the question arises as to how metabolic reprogramming can be integrated into a therapeutic procedure to cancer.

Melanoma is a malignant invasive tumor, and the global incidence rate is increasing. The BRAFV600E mutation is found in approximately 50% of melanoma patients, facilitating the development of targeted drugs. Vemurafenib (Zelboraf; Plexxikon/Roche) is the first drug approved for the treatment of BRAF mutation metastatic melanoma in the United States in August 2011 and in the European Union in February 2012. Together with significant advances in immunotherapy, it has significantly improved the five-year survival rate of patients with metastatic melanoma. However, the long-term prognosis of patients with metastatic melanoma is still unsatisfactory, which is probably related to drug resistance, the major obstacle of cancer therapy [46]. Drug resistance is far from being sufficiently researched, as it is a multifactorial process involving a number of mechanisms in which cancer stem cells (CSCs) may play a crucial role [47–49].

CSCs are defined as cancer cells that exhibit certain characteristics of embryonic stem cells [50, 51], which give rise to the entire set of cell types from a single cell and high expression of OCT4, SOX2 and so forth stemness markers [52]. Besides, CSCs stably express high levels of the ATP-binding cassette (ABC) transporter family, such as ABCG2, which is an important transporter responsible for pumping out chemotherapeutic drugs [53, 54]. It facilitating that CSCs escaped from most conventional chemotherapeutic agents. Given the importance of cancer stem cells (CSCs) in tumor metastasis [55, 56], we ought to ask which enzymes are contribute to melanoma cell stemness and response to targeted therapy. Generally, we assessed the mRNA expression-based stem cell index (mRNAsi) of melanomas using the OCLR algorithm developed by Malta et al. [57]. The genes responsible for cholesterol uptake (LDLR), biosynthesis (SREBF2, HMGCR, and DHCR24), and efflux (ABCA1 and ABCG1) were selected and divided into groups of 25% and 25%, respectively, according to their own expression level in the TCGA-SKCM cohort. As shown in Supplementary material Fig. S2A, patients with activated cholesterol biosynthesis genes showed higher stemness indices, and those with higher expression of efflux genes (ABCA1, ABCG1) correlated with lower stemness score, whereas the groups with high and low LDLR expression were comparable. When looking at the Spearman correlation between the mRNAsi scores and the genes related to cholesterol metabolism, only DHCR24, ABCA1, and ABCG1 were shown to be statistical significance (Supplementary material Fig. S2A). The mRNAsi results showed that elevated intracellular cholesterol was positively associated with melanoma stemness, through activation of DHCR24 in cholesterol biosynthesis or inhibition of efflux by ABCA1, and ABCG1. In line with this, our tissue microarray showed that DHCR24 was upregulated in malignant melanomas compared with normal skin tissue and which was consistent with previous conclusions that DHCR24 expression was increased in metastatic melanomas and that high DHCR24 levels were associated with greater tumor cell growth ability [20]. We observed that forced expression of DHCR24 promoted tumor growth in mouse xenografts (Fig. S1C), whereas knockdown of DHCR24 blocked cells in S phase and resulted in significant inhibition of invasion and migration in melanoma cells. More importantly, DHCR24 reproduced the cholesterol phenotype by promoting the melanoma spheroid propagation, indicating the oncogenic role of DHCR24 in melanoma cells.

Targeting cholesterol metabolism is currently being used as a potential therapeutic approach for cancer therapy. Inhibition of mevalonate metabolism, silencing of the low-density lipoprotein receptor, and conjugation of cholesterol components with anticancer drugs have shown attractive effects on slowing cell growth and inducing apoptosis in various cancers [58–60]. However, cholesterol may not be the ultimate effector of cancer cell survival. Intermediate metabolites of cholesterol have been shown to have various functions in tumorigenesis. We and others have previously shown that 7-dehydrocholesterol and vitamin K3 inhibit melanoma cell proliferation and induce apoptosis via AKT /MAPK signaling pathways [61–63], while Nelson et al. found that the cholesterol metabolite 27HC promoted tumor growth and metastasis in mouse models of breast cancer by serving as a partial agonist for the estrogen receptor and liver X receptor [27]. Moreover, 27HC has been reported to promote cisplatin resistance in glioblastoma cells, and high 27HC concentration is associated with poor patient outcome[64]. Indeed, our data showed that forced expression of DHCR24 increases cellular 27HC content without statistically affecting cholesterol content in melanoma cells and, moreover, is associated with melanoma spheroid expansion and vemurafenib resistance. At the same time, our data show that DHCR24 induces CYP27A1 expression and increases HMGCR and CYP27A1 expression in vemurafenib-resistant melanoma cell lines, suggesting an enhanced regulatory loop mechanism. Therefore, we finally demonstrated that targeting CPY27A1 has a dramatic inhibitory effect on vemurafenib-resistant melanoma cells when used in combination with vemurafenib. We observed that mevastatin had a similar effect on slowing the proliferation of vemurafenib-resistant melanoma as dafadine-A. We believed that this was likely due to the fact that mevastatin targets HMGCR, resulting in the absence of a 27HC synthesis source.

Rap1 (Ras-like GTPases), the closest relative of the small GTPase Ras, was identified as a suppressor of Ras transformation in rodent fibroblasts in a tissue culture-based genetic screening assay [65]. Ishida et al. suggested that Rap1 is particularly important for the maintenance of normal myelopoiesis and may provide an oncogenic signal in cooperation with RAS/ERK signaling [66]. The posttranslational addition of a geranylgeranyl group to the processed Rap1 carboxyl terminus binds Rap1 to the membrane, including intracellular membranes in the perinuclear region, endocytic and exocytic vesicles, and the plasma membrane [67], suggesting a role for Rap1 in recruiting components for transport and activation of membrane-coupled receptor kinase. In this work, we found that DHCR24 and DHCR24-induced 27HC significantly upregulated Rap1A/Rap1B expression and further activated the PI3K/AKT signaling pathway in melanoma spheroids, while Rap1 agonists or AKT inhibitors reproduced the stem cell phenotypes. Interestingly, we observed that in vitro administration of 27HC to melanoma cells significantly increased membrane fluidity (Fig. S3A), suggesting activation of signaling transduction. Compared to wild-type melanoma cells, Rap1A/Rap1B expression remains unchanged upon treatment with vemurafenib, but is downregulated by combined treatment with a CYP27A1 inhibitor in vemurafenib-resistant melanoma cell lines, highlighting the potential of cholesterol metabolism in vemurafenib-resistant melanoma.

### Supplementary Information

Below is the link to the electronic supplementary material.

Supplementary file1 (DOCX 12285 KB)

## Data Availability

All the data and materials supporting the results presented in this paper are available upon reasonable request.

## References

[1] Brose MS, Volpe P, Feldman M (2002). BRAF and RAS mutations in human lung cancer and melanoma. Can Res.

[2] Ribas A, Gonzalez R, Pavlick A (2014). Combination of vemurafenib and cobimetinib in patients with advanced BRAFV600-mutated melanoma: a phase 1b study. Lancet Oncol.

[3] McArthur GA, Chapman PB, Robert C (2014). Safety and efficacy of vemurafenib in BRAFV600E and BRAFV600K mutation-positive melanoma (BRIM-3): extended follow-up of a phase 3, randomised, open-label study. Lancet Oncol.

[4] Hauschild A, Grob J-J, Demidov LV (2012). Dabrafenib in BRAF-mutated metastatic melanoma: a multicentre, open-label, phase 3 randomised controlled trial. The Lancet.

[5] Flaherty KT, Infante JR, Daud A (2012). Combined BRAF and MEK inhibition in melanoma with BRAF V600 mutations. N Engl J Med.

[6] Garbe C (2016). Diagnosis and treatment of melanoma. European consensus-based interdisciplinary guideline—update 2016. Eur J Cancer.

[7] Yan C, LanGuo C-Y, ShinjiUrata YoshishigeShao, Jiang-HuaLi T-S (2017). Doxorubicin-induced mitophagy contributes to drug resistance in cancer stem cells from HCT8 human colorectal cancer cells. Cancer Lett.

[8] Ping Z (2014). Cancer stem cell and drug resistance. J Oral Maxillofac Surg.

[9] Ehmsen S, Pedersen MH, Wang G (2019). Increased cholesterol biosynthesis is a key characteristic of breast cancer stem cells influencing patient outcome. Cell Rep.

[10] Wang C, Li P, Xuan J (2017). Cholesterol enhances colorectal cancer progression via ROS elevation and MAPK signaling pathway activation. Cell Physiol Biochem.

[11] Qin Y, Hou Y, Liu S (2021). A Novel Long Non-coding RNA lnc030 maintains breast cancer stem cell stemness by stabilizing SQLE mRNA and increasing cholesterol synthesis. Adv Sci.

[12] García-Jiménez C, Gutiérrez-Salmerón M, Chocarro-Calvoet A (2016). From obesity to diabetes and cancer: epidemiological links and role of therapies. Br J Cancer.

[13] Gross S, Hooper R, Tomar D (2022). Suppression of Ca(2+) signaling enhances melanoma progression. EMBO J.

[14] Mirza R, Hayasaka S, Takagishi Y (2006). DHCR24 gene knockout mice demonstrate lethal dermopathy with differentiation and maturation defects in the epidermis. J Investig Dermatol.

[15] Waterham HR, Koster J, Romeijn GJ (2001). Mutations in the 3β-hydroxysterol Δ24-reductase gene cause desmosterolosis, an autosomal recessive disorder of cholesterol biosynthesis. Am J Hum Genet.

[16] Crameri A, Biondi E, Kuehnle K (2006). The role of seladin-1/DHCR24 in cholesterol biosynthesis, APP processing and Abeta generation in vivo. EMBO J.

[17] Zhang WB, Huang Y, Guo XR (2023). DHCR24 reverses Alzheimer's disease-related pathology and cognitive impairment via increasing hippocampal cholesterol levels in 5xFAD mice. Acta Neuropathol Commun.

[18] Cooper MK (1998). Teratagon-mediated inhibition of target tissue response to Shh signaling. Science.

[19] Qiu T, Cao J, Chenet W (2020). 24-Dehydrocholesterol reductase promotes the growth of breast cancer stem-like cells through the Hedgehog pathway. Cancer Sci.

[20] Di Stasi D, Vallacchi V, Campi V (2005). DHCR24 gene expression is upregulated in melanoma metastases and associated to resistance to oxidative stress-induced apoptosis. Int J Cancer.

[21] Tian W, Pang W, Ge Y (2018). Hepatocyte-generated 27-hydroxycholesterol promotes the growth of melanoma by activation of estrogen receptor alpha. J Cell Biochem.

[22] Battista MC, Guimond MO, Robergeet C (2010). Inhibition of DHCR24/Seladin-1 impairs cellular homeostasis in prostate cancer. Prostate.

[23] Bonaccorsi L, Luciani P, Nesi G (2008). Androgen receptor regulation of the seladin-1/DHCR24 gene: altered expression in prostate cancer. Lab Invest.

[24] Swinnen JV, Brusselmans K, Verhoeven G (2006). Increased lipogenesis in cancer cells: new players, novel targets. Curr Opin Clin Nutr Metab Care.

[25] Silvente-Poirot S, Poirot M (2014). Cholesterol and cancer, in the balance. Science.

[26] Asghari A, Umetani M (2020). Obesity and cancer: 27-hydroxycholesterol, the missing link. Int J Mol Sci.

[27] Nelson ER, Wardell SE, Jasper JS (2013). 27-Hydroxycholesterol links hypercholesterolemia and breast cancer pathophysiology. Science.

[28] Raza SS (2016). Role of cholesterol metabolite, 27-hydroxycholesterol in breast and prostate cancer. Int Immunopharmacol.

[29] Marwarha G, Raza S, Hammer K (2017). 27-hydroxycholesterol: a novel player in molecular carcinogenesis of breast and prostate cancer. Chem Phys Lipids.

[30] Umetani M, Shaul PW (2011). 27-Hydroxycholesterol: the first identified endogenous SERM. Trends Endocrinol Metab.

[31] DuSell CD, Umetani M, Shaulet PW (2008). 27-Hydroxycholesterol is an endogenous selective estrogen receptor modulator. Mol Endocrinol.

[32] Raza S, Meyer M, Schommer J (2016). 27-Hydroxycholesterol stimulates cell proliferation and resistance to docetaxel-induced apoptosis in prostate epithelial cells. Med Oncol.

[33] Alfaqih MA, Nelson ER, Liu W (2017). CYP27A1 loss dysregulates cholesterol homeostasis in prostate cancer. Can Res.

[34] Olsson M, Gustafsson O, Skogastierna C (2007). Regulation and expression of human CYP7B1 in prostate: overexpression of CYP7B1 during progression of prostatic adenocarcinoma. Prostate.

[35] Li D, Long W (2018). 27-hydroxycholesterol inhibits sterol regulatory element-binding protein 1 activation and hepatic lipid accumulation in mice. Obesity.

[36] Keung EZ, Gershenwald JE (2018) The eighth edition American Joint Committee on Cancer (AJCC) melanoma staging system: implications for melanoma treatment and care*.* Expert Rev Anticancer Ther 18(8): 775–784 10.1080/14737140.2018.1489246 PMC7652033 29923435

[37] Okuno K, Xu C, Pascual-Sabater S (2022). Berberine overcomes gemcitabine-associated chemoresistance through regulation of Rap1/PI3K-Akt signaling in pancreatic ductal adenocarcinoma. Pharmaceuticals (Basel).

[38] Hutchinson J, Marignol L (2017). Clinical potential of statins in prostate cancer radiation therapy. Anticancer Res.

[39] Huang BZ, Chang JI, Li E (2017). Influence of statins and cholesterol on mortality among patients with pancreatic cancer. JNCI.

[40] Kuzu OF, Noory MA, Robertson GP (2016). The role of cholesterol in cancer. Can Res.

[41] Xu H, Xia H, Zhou S (2021). Cholesterol activates the Wnt/PCP-YAP signaling in SOAT1-targeted treatment of colon cancer. Cell Death Discov.

[42] Sheng R, Kim H, Lee H (2014). Cholesterol selectively activates canonical Wnt signalling over non-canonical Wnt signalling. Nat Commun.

[43] Ma L, Cho W, Nelson ER (2022). Our evolving understanding of how 27-hydroxycholesterol influences cancer. Biochem Pharmacol.

[44] Wu Q, Ishikawa T, Sirianni R (2013). 27-hydroxycholesterol promotes cell-autonomous ER-positive breast cancer growth. Cell Rep.

[45] De Medina P, Paillasse MR, Segala G (2013). Dendrogenin A arises from cholesterol and histamine metabolism and shows cell differentiation and anti-tumour properties. Nat Commun.

[46] Shekhar MP (2013). Drug resistance: challenges to effective therapy. Curr Cancer Drug Targets.

[47] Saito Y, Kitamura H, Hijikata A (2010). Identification of therapeutic targets for quiescent, chemotherapy-resistant human leukemia stem cells. Sci Transl Med.

[48] Visvader JE, Lindeman GJ (2008). Cancer stem cells in solid tumours: accumulating evidence and unresolved questions. Nat Rev Cancer.

[49] Liu R, Wang X, Chen GY (2007). The prognostic role of a gene signature from tumorigenic breast-cancer cells. N Engl J Med.

[50] Ho MM, Ng AV, Lamet S (2007). Side population in human lung cancer cell lines and tumors is enriched with stem-like cancer cells. Can Res.

[51] Takubo K, Suda T (2012). Roles of the hypoxia response system in hematopoietic and leukemic stem cells. Int J Hematol.

[52] Mathieu J, Zhang Z, Zhouet W (2011). HIF induces human embryonic stem cell markers in cancer cells. Can Res.

[53] Zhang M, Mathur A, Zhanget Y (2012). Mithramycin represses basal and cigarette smoke-induced expression of ABCG2 and inhibits stem cell signaling in lung and esophageal cancer cells. Can Res.

[54] Lou H, Dean M (2007). Targeted therapy for cancer stem cells: the patched pathway and ABC transporters. Oncogene.

[55] Marta PV, Ryou-U T, Wataru U (2017). Drug resistance driven by cancer stem cells and their niche. Int J Mol Sci.

[56] Najafi M, Mortezaee K, Majidpoor J (2019). Cancer stem cell (CSC) resistance drivers. Life Sci.

[57] Malta TM, Sokolov A, Gentleset AJ (2018). Machine learning identifies stemness features associated with oncogenic dedifferentiation. Cell.

[58] Clendening J, Penn L (2012). Targeting tumor cell metabolism with statins. Oncogene.

[59] Scully T, Kase N, Gallagher EJ (2020). SAT-151 Regulation of low-density lipoprotein receptor expression in triple negative breast cancer. J Endocrine Soc.

[60] Albuquerque HMT, Santos CMM, Silva AMS (2018). Cholesterol-based compounds: recent advances in synthesis and applications. Molecules.

[61] Liu J, Zhong F, Caoet L (2020). 7-dehydrocholesterol suppresses melanoma cell proliferation and invasion via Akt1/NF-κB signaling. Oncol Lett.

[62] Gelzo M, Granato G, Albanoet F (2014). Evaluation of cytotoxic effects of 7-dehydrocholesterol on melanoma cells. Free Radical Biol Med.

[63] Shah M, Stebbins JL, Dewing A (2009). Inhibition of Siah2 ubiquitin ligase by vitamin K3 (menadione) attenuates hypoxia and MAPK signaling and blocks melanoma tumorigenesis. Pigment Cell Melanoma Res.

[64] Liu L, Li MY, Xinget Y (2019). The oncogenic roles of 27-hydroxycholesterol in glioblastoma. Oncol Lett.

[65] Kitayama H, Sugimoto Y, Matsuzakiet T (1989). A ras-related gene with transformation suppressor activity. Cell.

[66] Ishida D, Kometani K, Yang H (2003). Myeloproliferative stem cell disorders by deregulated Rap1 activation in SPA-1-deficient mice. Cancer Cell.

[67] Pizon V, Desjardins M, Bucci C (1994). Association of Rap1a and Rap1b proteins with late endocytic/phagocytic compartments and Rap2a with the Golgi complex. J Cell Sci.

