# Supplementary material for: Cholesterol neutralized vemurafenib treatment by promoting melanoma stem-like cells via its metabolite 27-hydroxycholesterol
Source: Cell Mol Life Sci. 2024 May 22;81(1):226. doi: 10.1007/s00018-024-05267-3 (PMC11111659; doi:10.1007/s00018-024-05267-3)
Supplement: Supplementary file 1 — Supplementary file1 (DOCX 12285 KB) [file 18_2024_5267_MOESM1_ESM.docx]

# Supplementary Files

**
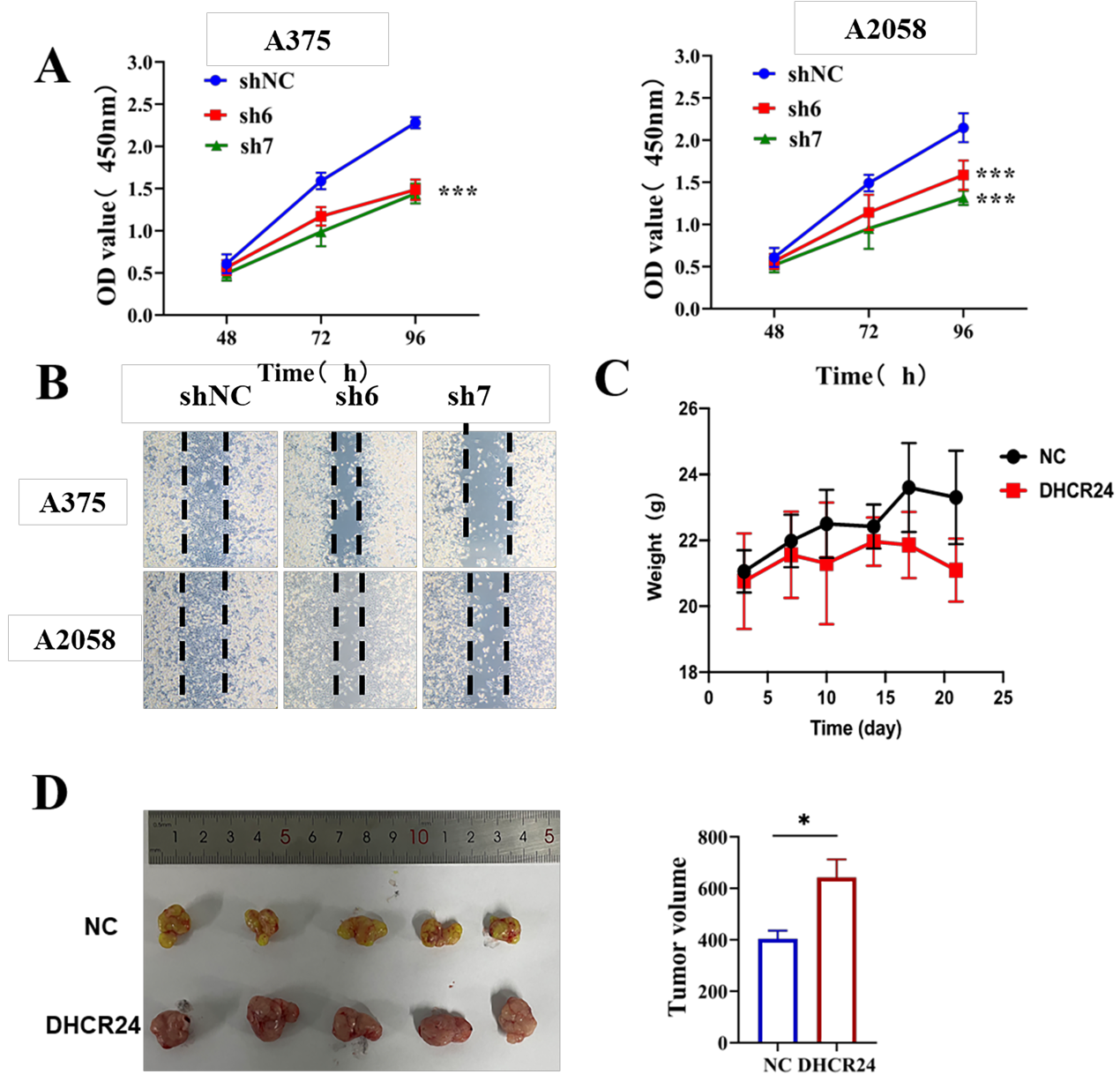
**

Fig.S1. Phenotypes of DHCR24 perturbation *in vitro* and *in vivo*. (A) Proliferation curves of melanoma cell A375 and A2058 were determined at 48, 72 and 96 hours after infection by lentivirus harboring scramble shRNA (shNC) and DHCR24 shRNAs (sh6 and sh7) using CKK-8. (B) Wound heal assay of A375 and A2058 melanoma cells harboring scramble shRNA (shNC) and DHCR24 shRNAs (sh6 and sh7). (C) The body weight of mice after A2058 xenografts. *P < 0.05. (D) Subcutaneous tumors generated in BALB/c-nu/nu mice, with DHCR24 or empty control (NC) transduced A2058 cells.


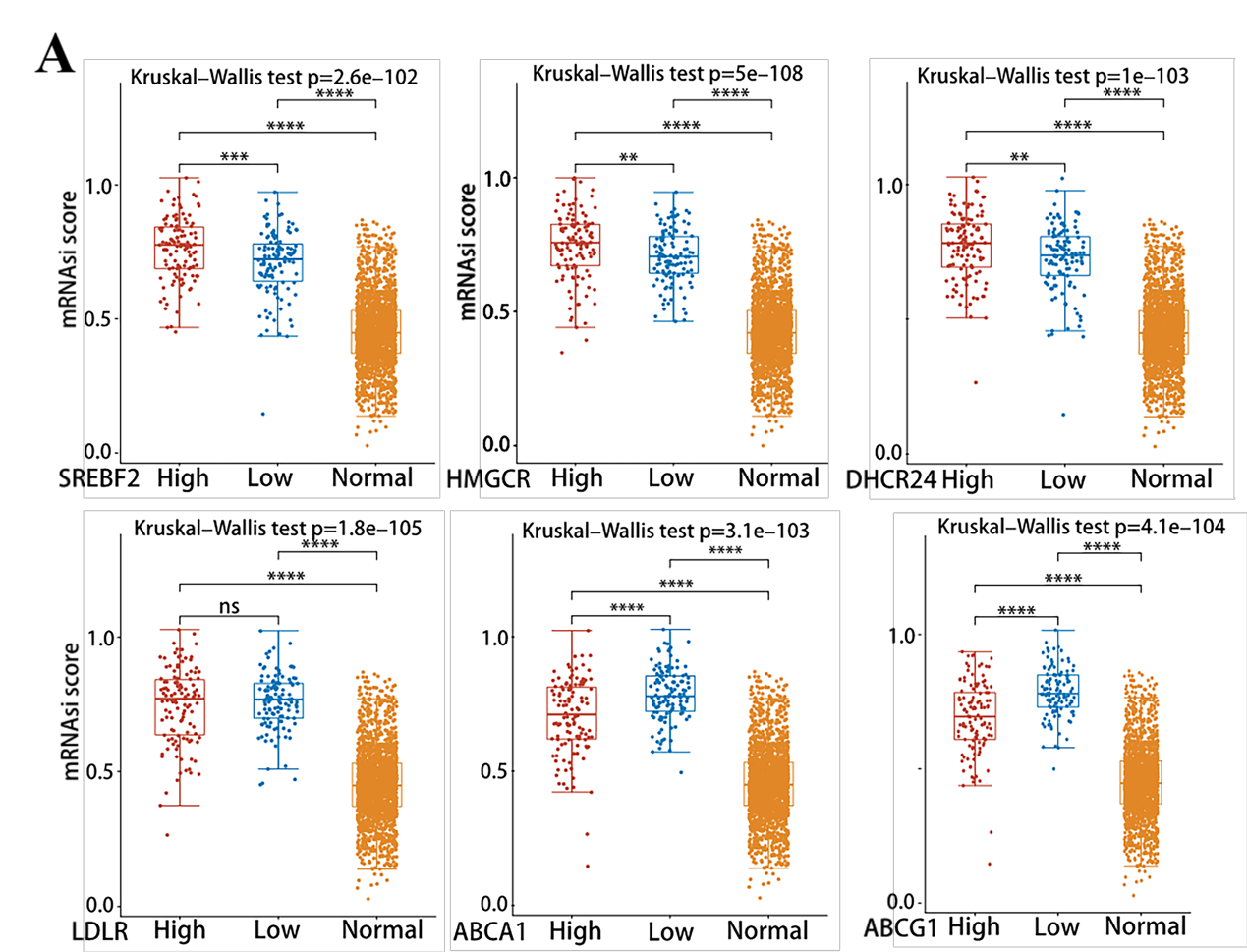


Fig.S2. Evaluation of melanoma stemness and cholesterol metabolism genes. (A) The distribution of OCLR scores in different groups. The abscissa represents different groups of samples, and the ordinate represents the distribution of the OCLR score. Different colors represent different groups, top-left represents the significance p-value test method. *p < 0.05, **p < 0.01, ***p < 0.001, asterisks (*) stand for significance levels. The statistical difference of two groups was compared through the Wilcox test, significance difference of three groups was tested with Kruskal-Wallis test.


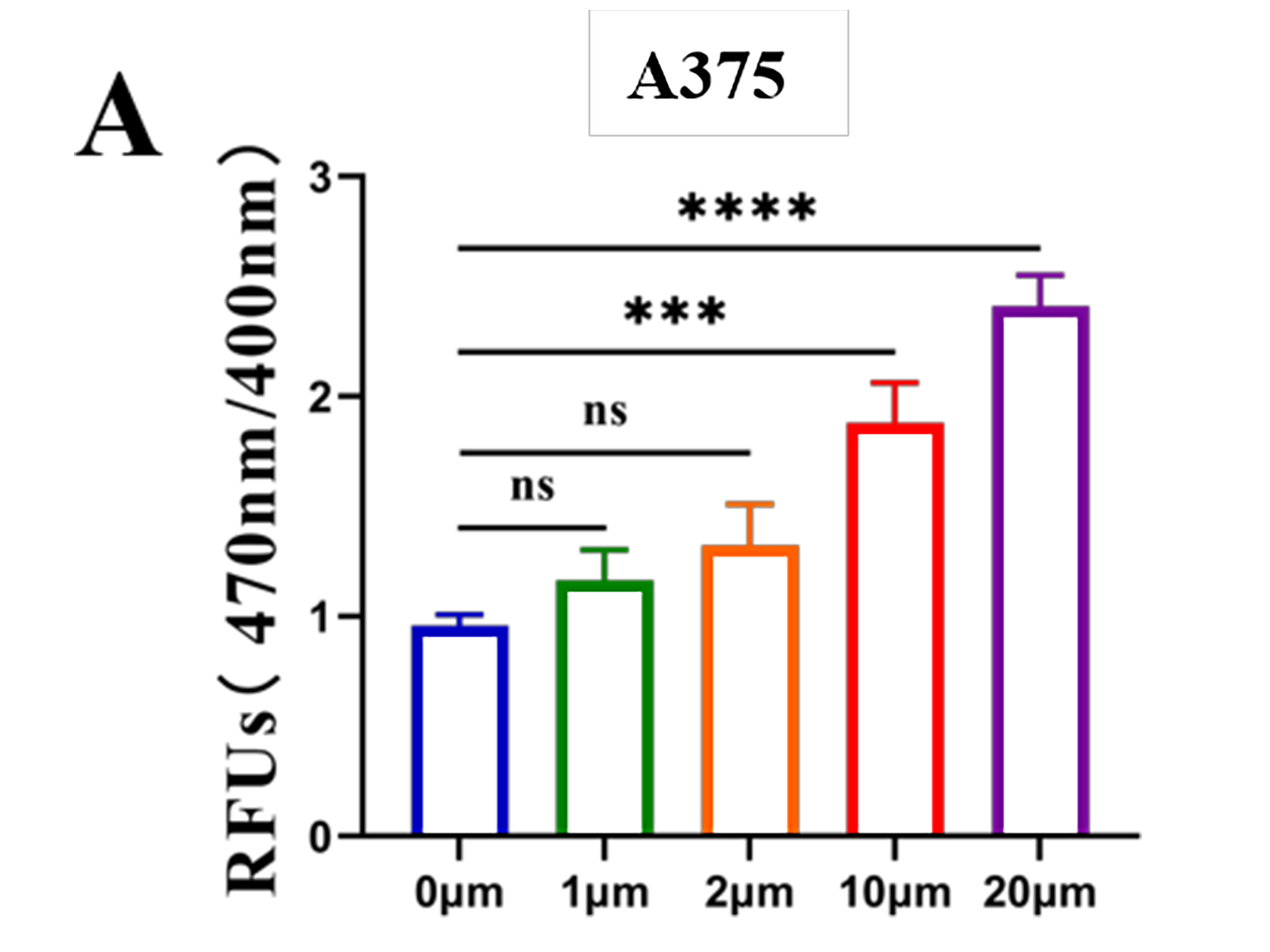


Fig.S3. Membrane fluidity evaluation. (A) Membrane fluidity was determined in A375 melanoma cell treated by concentration gradient 27HC.
